# Direct anterior approach for total hip arthroplasty with a novel mobile traction table -a prospective cohort study

**DOI:** 10.1186/s12891-017-1427-2

**Published:** 2017-01-31

**Authors:** Junichi Nakamura, Shigeo Hagiwara, Sumihisa Orita, Ryuichiro Akagi, Takane Suzuki, Masahiko Suzuki, Kazuhisa Takahashi, Seiji Ohtori

**Affiliations:** 1 Department of Orthopaedic Surgery, Graduate School of Medicine, Chiba University, 1-8-1 Inohana, Chuo-ku, Chiba City, Chiba 260-8677 Japan; 2 Department of Bioenvironmental Medicine, Graduate School of Medicine, Chiba University, 1-8-1 Inohana, Chuo-ku, Chiba City, Chiba 260-8677 Japan; 3 Research Center for Frontier Medical Engineering, Chiba University, 1-33 Yayoi-cho, Inage-ku, Chiba City, 263-8522 Japan

**Keywords:** Mobile traction table, Total hip arthroplasty, Direct anterior approach in supine position

## Abstract

**Background:**

The purpose of this prospective cohort study was to clarify the safety and efficacy of total hip arthroplasty via the direct anterior approach in the supine position with a novel mobile traction table.

**Methods:**

The first experience of consecutive surgeries by a single surgeon using the direct anterior approach with a traction table is described with a two-year follow-up period. Of 121 patients, 100 patients without previous hip surgeries, severe deformity, or cemented implants were divided into two groups comprising the first 50 patients and the second 50 patients.

**Results:**

The implant survival rate was 99% at the two-year follow-up. Revision surgery was required for periprosthetic femoral fracture in one patient. The complication rate possibly related to the traction table was 5% (5 patients): three anterior dislocations, one periprosthetic femoral fracture, and one intraoperative perforation caused by femoral rasping. The complication rate tended to decrease in the second group compared to the first group (4% versus 6%). Mean surgical time (72.0 minutes versus 82.5 min, *p* = 0.027), rate of allogeneic blood transfusion (2% versus 24%, *p* = 0.001), and cup alignment in the safe zone (100% versus 88%, *p* = 0.027) were significantly improved in the second group compared to the first group.

**Conclusion:**

The direct anterior approach with a novel mobile traction table showed a positive learning curve for surgical time, rate of allogeneic blood transfusion, and cup alignment in the safe zone.

## Background

Total hip arthroplasty (THA) is a successful treatment for painful hip disorders. The direct anterior approach (DAA) for this surgery has become of great interest because of its quicker recovery and lower dislocation rate over the last decade [1–3]. DAA is a muscle sparing approach that separates both the true inter-muscular and inter-nervous planes to reach the hip joint using the distal part of the Smith-Petersen approach (Hueter approach) [4, 5]. Theoretically, DAA is the most minimally invasive technique for hip surgery and is gaining in popularity [6]. On the other hand, DAA is technically demanding. The traction table, initially proposed by Judet et al. [7], assists the surgery by providing efficient exposure of the hip joint [8–11]. Fluoroscopy also guides implant position and insertion [12, 13]. However, Steiger RN et al. indicated that only 20% of orthopedic surgeons in Australia have mastered DAA with a traction table (traction DAA) [14]. Moreover, an earlier fixed-type special traction table is large and expensive for those just starting to use DAA. An alternative technique for DAA is manual leg control using a standard surgical table [15–18]. Homma et al. [18] suggested that manual leg control with a standard surgical table was sufficient for DAA, although the rate at which surgeons have discontinued the procedure is unknown. Besides, the direction of a surgical table is structurally opposite (180°) for the traction table, and for manual leg control. Because of the different direction, an incorrect table setting could cause a serious problem with femoral exposure using the manual leg control approach as this procedure uses a bending function of the surgical table to extend the hip joints around the buttocks. Thirdly, the fixed type traction table is large and expensive for a surgeon inexperienced with DAA. Therefore, we developed a novel mobile traction table to facilitate traction DAA. The table is less expensive and saves operating room space. The purpose of this prospective cohort study was to clarify the safety and efficacy of THA via DAA in the supine position with a novel mobile traction table.

## Methods

The research protocol of this prospective cohort study was in compliance with the Helsinki Declaration, approved by the Institutional Review Boards, and registered with the University Hospital Medical Information Network. Written informed consent was obtained from all study participants. The inclusion criterion was primary THA; all THAs for which the first author was not the surgeon were excluded. From May 2012 to April 2014, 121 primary THAs were performed on consecutive patients in our institute using traction DAA. The first author operated on 114 patients, and less experienced surgeons operated on seven patients who were not included in this study. Fourteen patients were regarded as the refractory group: five patients with previous hip surgeries, five patients with severe osteoporosis requiring cemented implants, and four patients with severe deformity requiring special stems. The remaining 100 patients (18 male and 82 female, 61 right and 39 left) were regarded as the standard group without previous hip surgeries, severe deformity, or cemented implants. The standard group of 100 patients was divided into two groups consisting of the first 50 patients and the second 50 patients to undergo surgery. Baseline patient characteristics of the standard group and the refractory group are shown in Table 1. Characteristics of the two groups within the standard group are shown in Table 2. One or more comorbidities were noted in 67 of 114 patients (59%). Physical status was classified using the American Society of Anesthesiologists classification [19]. In the standard group, cementless, rectangular cross section type, straight-stems were used, with a highly cross-linked polyethylene cementless cup, and cobalt-chrome femoral head. In the refractory group, 4 patients used this same combination, but 5 patients used cementless, cylindrical modular type, straight-stems to adjust for excessive anteversion. The other 5 patients used cemented, polished, double tapered, rectangular straight-stems, with a cementless cup in two patients and a cemented cup in three patients.

**Table 1:** Comparison of the standard group and the refractory group

	Standard group (*n* = 100)	Refractory group (*n* = 14)	*p* values
Gender (Male: Female)	18:82	5:9	0.154
Age	62.3 ± 13.7	66.8 ± 7.7	0.231
Diagnosis (OA:ION:RA)	62:32:6	10:1:3	0.039^b^
ASA-PS (I:II:III)	37:44:19	2:5:7	0.027^b^
BMI	23.6 ± 3.8	23.7 ± 4.1	0.903
Preoperative Hb	12.6 ± 1.5	12.3 ± 1.4	0.411
Preoperative JOA hip score	38.8 ± 13.4	38.1 ± 17.6	0.864
Surgical time (minutes)	77.3 ± 23.7	115.1 ± 40.7	0.001^a^
Intraoperative blood loss (ml)	256 ± 186	506 ± 558	0.001^a^
Estimated total blood loss (ml)	835 ± 340	1084 ± 649	0.026^a^
Allogeneic blood transfusion	13 (13%)	5 (36%)	0.045^b^
Cup abduction angle (degrees)	41.0 ± 4.1	42.2 ± 7.9	0.388
Cup anteversion (degrees)	16.8 ± 5.1	18.5 ± 6.9	0.254
Cup safe zone	94 (94%)	11 (79%)	0.080
Stem varus angle (degrees)	0.1 ± 0.5	0.0 ± 0.0	0.494
Stem flexion angle (degrees)	0.6 ± 1.0	0.3 ± 0.7	0.262
Stem safe zone	94 (94%)	14 (100%)	1.000
Leg length discrepancy (mm)	6.4 ± 9.0	5.4 ± 7.4	0.673
Postoperative JOA hip score			
At three months	84.4 ± 9.4	77.9 ± 7.6	0.014^a^
At two years	87.8 ± 8.2	83.6 ± 6.1	0.066
Surgical complication (category 2 and 3)	5 (5%)	0 (0%)	1.000
Revision	1 (1%)	0 (0%)	1.000

*OA* osteoarthritis, *ION* idiopathic osteonecrosis, *RA* rheumatoid arthritis, *ASA-PS* American Society of Anesthesiologists-Physical status, *BMI* body mass index, *Hb* hemoglobin, *JOA* Japanese Orthopedic Association. Mean ± standard deviation

^a^Student’s t-tests and ^b^Pearson *χ* ^2^ tests

**Table 2:** Comparison of the first 50 cases and the second 50 cases in the standard group

	First 50 cases	Second 50 cases	*p* values
Gender (Male:Female)	7:43	11:39	0.436
Age	64.5 ± 13.4	60.1 ± 13.7	0.110
Diagnosis (OA:ION:RA)	30:16:4	32:16:2	0.694
ASA-PS (I:II:III)	17:20:13	20:24:6	0.203
BMI	23.8 ± 3.8	23.4 ± 3.8	0.674
Preoperative Hb	12.3 ± 1.4	13.0 ± 1.5	0.020^a^
Preoperative JOA hip score	34.9 ± 12.2	42.8 ± 13.5	0.003^a^
Surgical time (minutes)	82.5 ± 27.1	72.0 ± 18.6	0.027^a^
Intraoperative blood loss (ml)	263 ± 172	249 ± 201	0.704
Estimated total blood loss (ml)	866 ± 361	803 ± 318	0.359
Allogeneic blood transfusion	12 (24%)	1 (2%)	0.001^b^
Cup abduction angle (degrees)	41.9 ± 4.5	40.1 ± 3.6	0.032^a^
Cup anteversion (degrees)	17.7 ± 6.0	15.8 ± 3.9	0.070
Cup safe zone	44 (88%)	50 (100%)	0.027^b^
Stem varus angle (degrees)	0.1 ± 0.8	0.1 ± 0.6	0.888
Stem flexion angle (degrees)	0.5 ± 1.1	0.7 ± 0.9	0.165
Stem safe zone	47 (94%)	47 (94%)	1.000
Leg length discrepancy (mm)	5.0 ± 5.7	7.9 ± 11.2	0.109
Postoperative JOA hip score			
At three months	85.1 ± 10.4	83.7 ± 8.3	0.478
At two years	89.0 ± 8.0	86.7 ± 8.3	0.176
Surgical complication (category 2 and 3)	3 (6%)	2 (4%)	1.000
Revision	1 (2%)	0 (0%)	0.999

*OA* osteoarthritis, *ION* idiopathic osteonecrosis, *RA* rheumatoid arthritis, *ASA-PS* American Society of Anesthesiologists-Physical status, *BMI* body mass index, *Hb* hemoglobin, *JOA* Japanese Orthopedic Association. Mean ± standard deviation

^a^Student’s t-tests and ^b^Pearson *χ* ^2^ tests

The first author had 8 years of surgical experience as an adult hip surgeon. He used the direct lateral approach in the lateral decubitus position in 61 patients for the first 2 years, the anterolateral (modified Watson-Jones) approach in the lateral decubitus position in 53 patients the following year and a half, and then used the direct anterior approach in the supine position without a traction table in 6 patients. He learned the direct anterior approach from Dr. Oinuma at Funabashi Orthopedic Hospital (Japan). After that, he introduced the traction table for this approach. Therefore, the first author had previous experience with 120 total hip arthroplasties at the beginning of the study.

### Surgical procedure

**Fig. 1:**
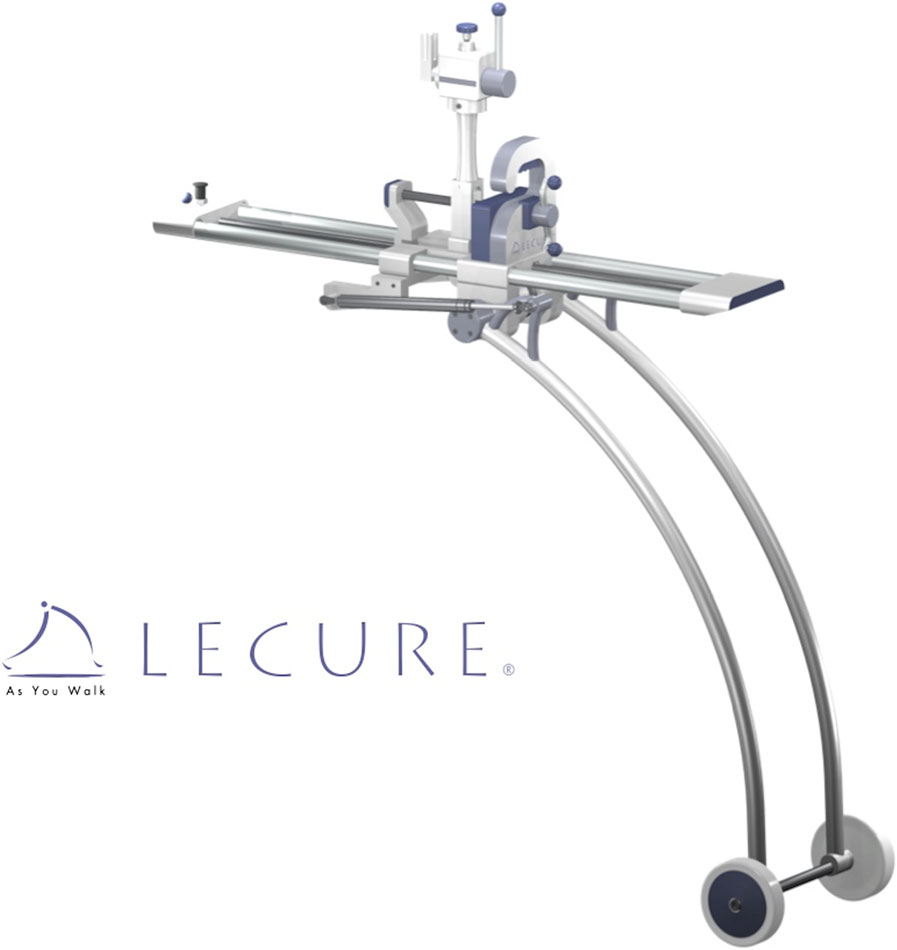
~As You Walk ~ LECURE ®, a novel mobile traction table

**Fig. 2:**
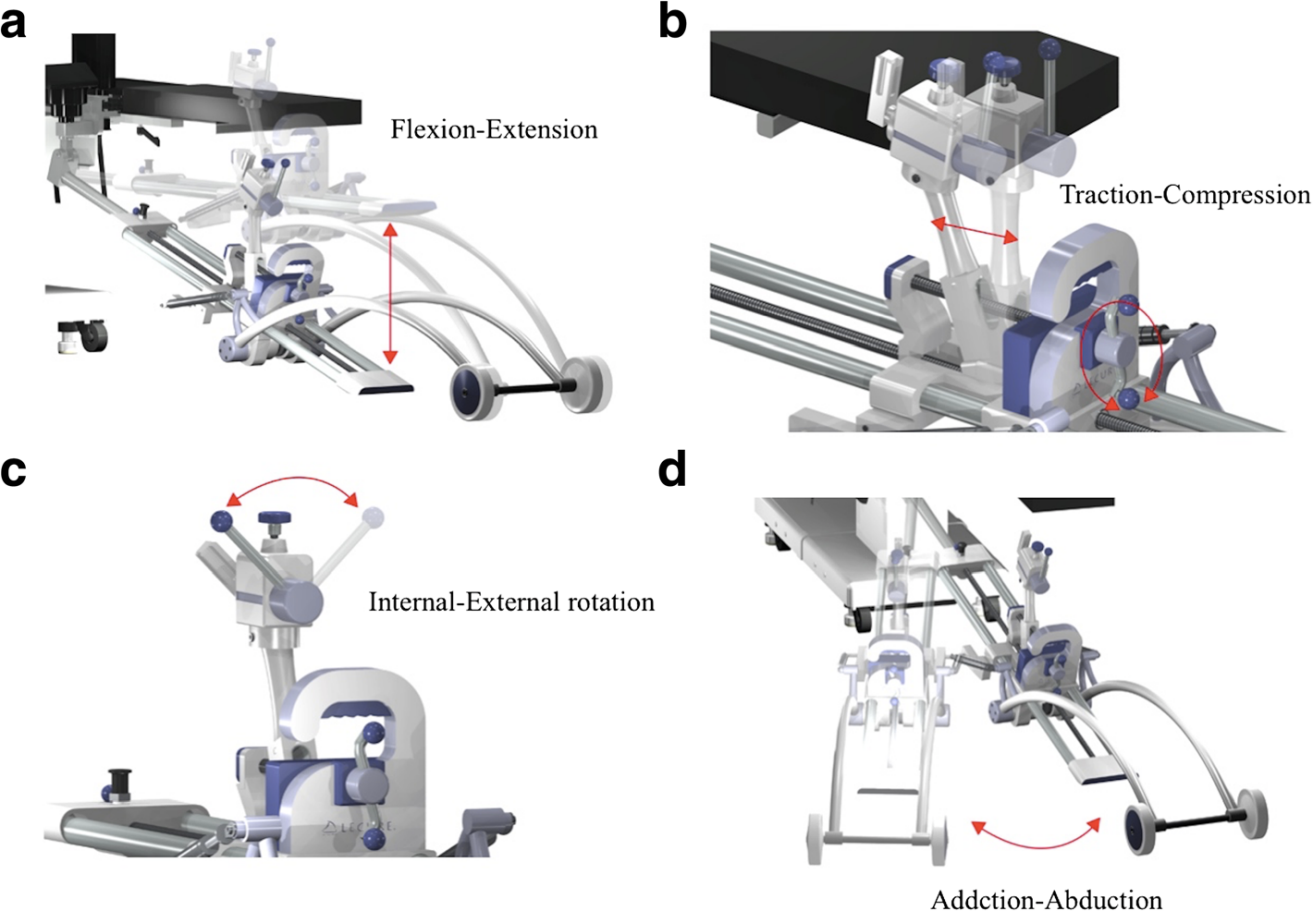
Movement of the mobile traction table. This table can hold the leg in hip flexion/extension (**a**), traction, distraction, or compression (**b**), internal/external rotation (**c**), or adduction/abduction (**d**)

Preoperative planning was based on computed tomography-assisted, three dimensional software (ZedHip, LEXI, Tokyo, Japan). After induction of general anesthesia, 1 g of tranexamic acid was intravenously injected before skin incision. Traction DAA for THA was performed with the patient in a supine position, lying on a novel mobile traction table, ~As You Walk ~ LECURE ® (Surgical Alliance, Tokyo, Japan) (Fig. 1). This table can be set up with a standard orthopedic surgical table, and can hold the leg in hip flexion/extension, internal/external rotation, adduction/abduction, or traction, distraction, or compression (Fig. 2). A non-scrubbed and non-sterile assistant handled the traction table. The table provides wide exposure of the proximal femur and the acetabulum both with direct visualization and fluoroscopy. The leather boot is designed to fit the foot and ankle snugly with a double locking bandage mechanism to avoid slipping off the foot. From the superior anterior iliac spine, Heuter’s interval between the tensor fascia lata and sartorius muscle is palpated. The skin incision runs along the mid-line of the tensor fascia lata. This line links the anterior superior iliac spine and the lateral femoral condyle and is more than one finger’s breadth from Heuter’s interval, avoiding injury of the lateral femoral cutaneous nerve. The incision begins 3 cm proximal from the tip of the greater trochanter and 9 cm distal to the trochanter (12 cm long in total). With fluoroscopy, a vertical line passing though the tip of the greater trochanter is identified and the pelvic tilt is adjusted by symmetry of the obturator foramina, centering the coccyx with the symphysis.

The sheath of the tensor fascia lata is incised longitudinally in the direction of the muscle fibers. The muscle fibers of the tensor fascia lata are intentionally pulled laterally within the sheath by the surgeon’s index finger and relaxed by exposing the intermuscular space toward the anterior superior iliac spine. The deep fascia of the rectus femoris is identified by white tendinous fibers, muscle fibers as red, and fat tissue as yellow (tricolor sign). A wound retractor is utilized without a surgical arm or a sinker to hold the retractor. After hemostasis of the arteriole branch, the deep fascia is incised longitudinally at the boundary of the fat tissue to spare the rectus femoris within the sheath. The femoral head is outlined by two narrow Homann retractors, one at the piriformis fossa and the other at the medial aspect of the femoral neck. The triangle of the anterior capsule is visualized; the medial line is the inferior iliofemoral ligament, the lateral line is the insertion of the vastus lateralis muscle and the capsule, and the superior line is the superior iliofemoral ligament. After capsulectomy, the upper outline of the cervicotrochanteric junction is visualized, then 45° of external rotation makes the lower outline clear, and mild traction causes subluxation. The osteotomy is performed in situ, perpendicular to the anterior inter-trochanteric plane. With traction, the osteotomy site is spontaneously opened and the femoral head can be easily removed. The posterior capsule between the acetabulum and the greater trochanter (ischiofemoral ligament and superior iliofemoral ligament) is resected. A blunt forked retractor is placed at the recess of the posterior wall of the acetabulum.

Femoral preparation is recommended before acetabular reaming (femoral first technique). Elevating the calcar femorale with a blunt hook, a sharp fork retractor is inserted into the posterolateral aspect of the greater trochanter. To avoid a hinge phenomenon of the greater trochanter at the posterior wall of the acetabulum, it is useful to apply internal rotation in the neutral position first, and then to apply elevation and external rotation to 90° to visualize the calcar. The ischiofemoral ligament and the superior iliofemoral ligament need to be detached until sufficient anterior mobilization of the greater trochanter is obtained. The pubofemoral ligament also needs to be detached to obtain sufficient lateral mobilization of the calcar. Hyperextension of 35°, relaxation and compression, and adduction of 10° are applied with the traction table. This relaxation technique prevents excessive stretch of the femoral nerve and also assists femoral exposure. A Z-shaped canal finder (Tanaka Ika, Tokyo, Japan) is an essential tool to identify the axis of the medullary canal and anteversion of the calcar. The entry point of the canal finder is the piriformis fossa, avoiding flexion insertion or perforation of the femur. Varus insertion can be avoided by removing the cancellous bone of the lateral femoral canal with the shoulder of this canal finder. Femoral rasps are inserted sequentially.

Acetabular reaming is performed with a straight holder, avoiding excessive reaming of the anterior and posterior walls of the acetabulum. Fluoroscopy intermittently monitors the height and the depth of the reaming, and the cup position. In severely dysplastic hips, the outline for the reaming is prepared with a round chisel. Cup fixation is press-fit and screw fixation uses two 20 mm screws. Reduction is performed by internal rotation and traction. Alignment of the implant and leg length discrepancy are confirmed with fluoroscopy. Anterior stability is examined by external rotation of 90° with mild traction. The acetabular bone defect is reconstructed with morselized autograft. At closure, no suction drains are applied, the fascia of the tensor is sutured continuously but the skin is not sutured, rather it is coated with an adhesive agent (Dermabond, Ethicon, NJ). A water-proof wound dressing is wrapped with a compressive bandage for a few days to prevent hematoma. Postoperative rehabilitation begins on postoperative day one and the patients are allowed full weight bearing.

### Measurements and outcomes

The primary outcome was implant survival at the end point of conversion to revision surgery. The secondary outcomes were surgical complications, surgical time, and blood loss. Surgical complications were categorized as (1) complications definitely not related to the traction table, (2) complications that may be related to the traction table, and (3) complications related to the traction table. Stem sinking was defined as more than 3 mm of stem subsidence compared to an X-ray taken immediately after surgery; subsidence was evaluated by two authors. Excessive acetabular reaming was defined as an iatrogenic bone defect of the acetabular wall created by reaming and was evaluated by the surgeon intraoperatively. Cup migration was defined as more than 5° of change of cup alignment on adjusted pelvic tilt compared to an X-ray taken immediately after surgery; migration was evaluated by two authors. Blood loss consisted of intraoperative blood loss recorded by the anesthesiologists, blood transfusion, and estimated total blood loss [20]; Estimated total blood loss = Estimated blood volume × (preoperative hemoglobin - post operative-day one hemoglobin) / preoperative hemoglobin + autologous blood transfusion volume + allogeneic blood transfusion volume. Other outcomes were the safe zone for implant alignment [21], and the preoperative and postoperative Japanese Orthopedic Association (JOA) hip scores [22] at three months and two years. Lateral inclination of 40 +/- 10° and anteversion of 15 +/- 10° measured from an anteroposterior X-ray of the pelvis in the supine position were regarded as the safe zone for cup alignment by Lewinnek et al. [4]. Cup anteversion was calculated by measurement of the short diameter and long diameter of the ellipse as follows:

Cup anteversion = sin^−1^ (short diameter / long diameter)

As for stem alignment, the angle of the axis of the stem and the femur within +/- 3° both in the anteroposterior and lateral views was regarded as the safe zone [5]. The alignment was measured with Image J 1.45 s (National Institute of Health, USA). JOA hip score was assessed in an unblinded fashion, mainly by the first author at the outpatient clinic before, three months after, and two years after surgery.

### Statistical analysis

The first 50 cases were compared with the second 50 cases using Student’s t-tests, Pearson *χ* ^2^ tests, and logistic regression analysis with multivariate and a forward step-wise selection (SPSS 16.0, Chicago, Illinois). A *p*-value <0.05 was considered significant.

## Results

The implant survival rate was 99% in the 100 standard patients at the two-year follow-up; one revision surgery was required for a periprosthetic femoral fracture. The surgical complication rate possibly related to the traction table (categories 2 and 3) was 5%: three anterior dislocations, one periprosthetic femoral fracture, and one intraoperative perforation from femoral rasping. Mean surgical time (72.0 min versus 82.5 min, *p* = 0.027), rate of allogeneic blood transfusion (2% versus 24%, *p* = 0.001), and cup alignment in the safe zone (100% versus 88%, *p* = 0.027) were significantly improved in the second group compared to the first group (Table 2).

**Fig. 3:**
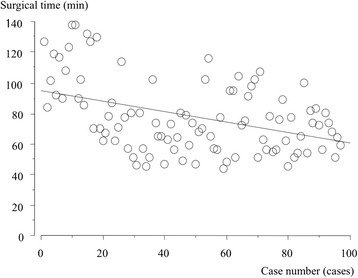
Surgical time for the initial 100 cases in the standard group. A learning curve is indicated by decreasing time by case. (Surgical time [min]) = 94.1 - 0.34 × (cases), R^2^ = 0.176, p = 0.001, simple regression analysis

**Fig. 4:**
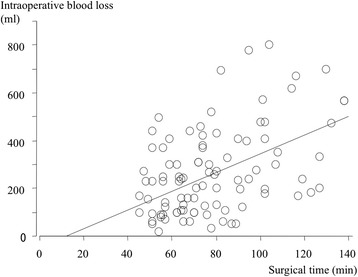
Intraoperative blood loss and surgical time in the standard group. (Intraoperative blood loss [ml]) = 3.9 × (surgical time [min]) - 47.7, R^2^ = 0.251, p = 0.001, simple regression analysis

**Fig. 5:**
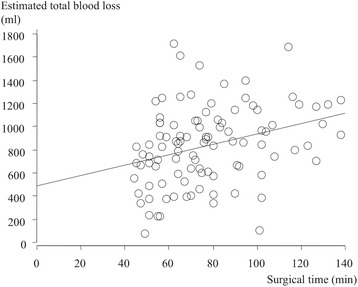
Estimated total blood loss and surgical time in the standard group. (Estimated total blood loss [ml]) = 4.5 × (surgical time [min]) + 487.4, R^2^ = 0.098, *p* = 0.002, simple regression analysis

**Fig. 6:**
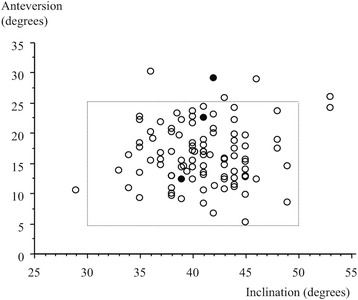
Scatterplot of cup inclination and anteversion in the standard group. The square outlined by the dotted lines reflects Lewinnek’s safe zone. The three black dots indicate dislocated hips

The surgical time was negatively correlated with the case number, with shorter surgical times as more cases were completed (Fig. 3). Both intraoperative and total estimated blood loss showed a positive correlation with the length of the surgery (Figs. 4 and 5). The cup inclination was 41.0° (range, 29° to 53°) and the anteversion was 16.8° (range, 5° to 30°) with 94% in the safe zone (Fig. 6). The preoperative JOA hip score significantly improved three months post-operatively, and continued to improve until two years both in the first group and the second group (*p* = 0.001, respectively, Table 2).

Category 1 surgical complications consisted of four sinking stems, two with excessive reaming of the acetabulum, two with cup migration, and one each with an insufficiency fracture of the ipsilateral calcaneus, the ipsilateral pubis, or the sacroiliac region. Category 2 consisted of one periprosthetic femoral fracture and one intraoperative perforation from femoral rasping. Category 3 consisted of three anterior dislocations. Seven patients died, but all deaths were unrelated to surgery: malignancy in four, cerebrovascular disease in two, and anaphylactic shock in one.

In the refractory group, longer surgical time and more blood loss was observed, and postoperative JOA hip score at three months was significantly lower than in the standard group (Table 1). However, the hip score was significantly improved at two years (*p* = 0.001).

Logistic regression analysis revealed that the rate of allogeneic blood transfusion decreased by 0.3 times for each 10 kg increase in patient weight and by 0.7 times with each decrease in surgical time by 10 min (*p* = 0.017), but increased 1.9 times for each 10 years of age (*p* = 0.031).

## Discussion

This study showed a moderate success rate for the first experience with traction DAA with an implant survival rate of 99% at the two-year follow-up. Our surgical complication rate of 5% was within the range of those reported in the literature for DAA (range: 0 - 24%, Table 3) [1–3, 5, 6, 8–13, 15–18, 23, 24]. Sariali et al. reported that only 57 of 1764 hips (3.2%) had complications, which occurred in the first 250 cases, indicating an initial complication rate of 22.8% [9]. In a multicenter cohort study of 1277 hips, Bhandari et al. documented a complication rate of 14.5%, but the rate was two times higher for surgeons who had performed less than 100 cases [10]. The 99% implant survival rate was consistent with previous reports, which ranged from 84.8% to 100% [1, 2, 6, 8, 10–13, 15–18, 23, 24]. Spaan et al. [24] reported the worst revision rate of 15.2% (7 of 46 hips) in their early experience with DAA with manual leg control, and abandoned the technique. Jewett et al. [12] and Woolson et al. [23] also reported a relatively high revision rate of about 3%. de Steiger et al. clearly showed an improvement in the cumulative percent revision at two years with surgical experience: 3 to 4% in the first 50 operations, 2% in operations 51-100, and 1% for surgeons doing more than 100 operations [14]. Therefore, we believe that our initial outcome is acceptable.

**Table 3:** Comparison of previous reports of THA via the DAA

Author name	Traction/Manual	Number of patients	Implant survival rate	Surgical complication rate	Surgical time (min)	Follow-up period (months)
Sariali^9^	Traction	1764	NA	3.2%	72	NA
Bhandari^10^	Traction	1277	97.3%	14.5%	95	NA
Siguier^2^	Traction	926	99.6%	1.9%	NA	NA
Jewett^12^	Traction	800	96.8%	15.8%	NA	21
Hartford^11^	Traction	500	98.4%	5.0%	NA	3
Matta^5^	Traction	494	NA	4.0%	75	NA
Woolson^23^	Traction	247	97.2%	15.4%	164	8
Laude^8^	Traction	80	98.8%	3.8%	55	NA
Barret^13^	Traction	43	100%	0%	84	12
Kennon^1^	Manual	672	100%	10.4%	60	6
Seng^17^	Manual	182	97.8%	2.2%	73	NA
Homma^18^	Manual	120	100%	0.8%	117	20
Rachbauer^3^	Manual	100	NA	11.0%	105	3
Oinuma^15^	Manual	99	100%	4.0%	79	17
Nakata^6^	Manual	99	100%	5.1%	105	6
Restrepo^16^	Manual	50	100%	0%	56	24
Spaans^24^	Manual	46	84.8%	23.9%	84	12

The superscript number of the author name reflects the reference number. *NA* not applicable

It is true that safety and accuracy take priority over speed and efficiency, but surgical time is also important. DAA is technically demanding due to its minimal invasiveness and limited exposure from the intermuscular plane. However, surgical time decreases with the surgeon’s experience. Previous reports showed that surgical time ranged from 55 min to 164 min [1, 3, 5, 6, 8–10, 13, 15–18, 23, 24]. In this study, surgical time declined from 83 min in the first 50 cases to 72 min in the second 50 cases. On the other hand, in refractory patients, 115 min were needed to complete the procedure. In the first author’s experience, mean surgical time was 157 min using the direct lateral approach, 123 min for the anterolateral approach, and 132 min for the direct anterior approach with manual leg positioning. This study showed decreased surgical time in traction DAA with a positive learning curve.

Implant alignment is key to stability and long-term retention. The cup safe zone rate of 94% in this study was acceptable in comparison with previous reports, which ranged from 27% to 99% [5, 6, 13, 18, 23, 25]. The stem safe zone rate of 94% also was comparable to previous reports, which ranged from 22% to 98% [6, 13, 15, 18, 25]. Homma et al [18] reported the lowest safe zone rate of 27% for the cup and Kobayashi et al. [25] reported the lowest safe zone rate of 22% for the stem in DAA with manual leg control, even using fluoroscopy. Our excellent success rate might be due to three dimensional planning-assisted implant positioning, sufficient soft tissue release and exposure of the surgical site, and adjustment of pelvic tilt using fluoroscopic guidance. Care should be taken to obtain accurate imaging due to the limited field of view in fluoroscopy.

The dislocation rate after DAA has been reported to be 0 - 2.2% including both anterior and posterior dislocation [1, 2, 5, 6, 8–10, 12, 13, 15, 17, 18, 23–25]. Of three dislocations in this study, two were even in the safe zone (Fig. 6), but hyperextension of the hip led to anterior dislocation. We insist that patient education is still essential for THA with muscle sparing techniques.

The advantage of DAA is muscle sparing of the gluteus medius and minimus as well as the gluteus maximus and the tensor fascia lata, which serve as hip abductors and pelvic stabilizers [5]. Preservation of the posterosuperior capsule or the short external rotators is also expected in DAA. Anatomically, the piriformis inserts on the tip of the greater trochanter, and the obturator internus and externus insert on the medial aspect of the greater trochanter, running anteriorly to the piriformis [26, 27]. The surgeon should pay attention to the tension on the leg during hip extension for femoral procedure, as too much tension may cause fracture of the trochanter or femoral nerve palsy. Matta et al. [5] suggested that stems requiring straight reamers for canal preparation are more difficult because they require the most anterior mobilization of the femur to allow access down the canal. If further mobilization of the femur is necessary, it can be accomplished with further release of the capsule and conjoint tendon and piriformis sequentially for sufficient exposure [28].

The role of the traction table has been controversial in DAA. Siguier et al. [2] and Matta et al. [5] used a special traction table for DAA. Siguier et al. [2] reported a 2% surgical complication rate in 926 THAs by traction DAA from 1993 to 2000, excluding obese patients, muscular male patients, and patients who had previous hip surgery or severely dysplastic hips. Matta et al. [5] reported 93% of implants aligned in the safe zone, a 4% surgical complication rate, and 75 min of surgical time in 494 THAs by traction DAA from 1996 to 2005. They used a classic Smith-Petersen approach before 2002 and a mini-incision after 2002. Kennon et al. [1] and Oinuma et al. [15] utilized a bending function with a standard surgical table and manual leg control. The two surgical procedures are similar, but traction DAA has several advantages. First, a perineal post stabilizes the pelvis and this stability allows reliable positioning of the acetabular reamer and component [2]. Second, fluoroscopy is easily used with a traction table to provide immediate feedback about the reaming process, and to verify accurate implant alignment [5]. Third, sterilization and draping are much easier, and sterilized assistants to hold the leg are not necessary, saving time, medical resources and human resources.

There are several limitations to this study. First, because the first author was an inexperienced surgeon with DAA, and also with the new traction table, improved safety and efficacy was due to a combination of these two factors, and could not just be attributed to the traction table. In other words, there was a learning curve for DAA as well as for the use of the traction table. de Steiger et al. reported that 50 or more operations were required to reach a revision rate similar to that of a high volume surgeon with traction DAA, based on an Australian registry [14]. Second, this study showed only a short-term outcome with a two-year follow-up period. However, the follow-up periods found in the literature were within two years, and so the long-term outcome of DAA is unknown (Table 3). A longer-term follow-up is necessary because Eto et al. reported that the mean duration from primary to revision THA was 3.0 years [29].

## Conclusions

The direct anterior approach with a novel mobile traction table showed a positive learning curve for surgical time, rate of allogeneic blood transfusion, and cup alignment in the safe zone.

## Abbreviations

DAA: Direct anterior approach
Hb: Hemoglobin
JOA: Japanese Orthopedic Association
THA: Total hip arthroplasty
traction DAA: DAA with traction table

## References

[1] Kennon RE, Keggi JM, Wetmore RS, Zatorski LE, Huo MH, Keggi KJ (2003). Total hip arthroplasty through a minimally invasive anterior surgical approach. J Bone Joint Surg Am.

[2] Siguier T, Siguier M, Brumpt B (2004). Mini-incision anterior approach does not increase dislocation rate: a study of 1037 total hip replacements. Clin Orthop Relat Res.

[3] Rachbauer F, Nogler M, Krismer M. Minimally invasive single-incision anterior approach for total hip arthroplasty -early results. Minimally Invasive Total Joint Arthroplasty. Berlin, Heidelberg: Springer-Verlag Berlin Heidelberg; 2004. p. 54–59.

[4] Smith-Petersen MN (1949). Approach to and exposure of the hip joint for mold arthroplasty. J Bone Joint Surg Am.

[5] Matta JM, Shahrdar C, Ferguson T (2005). Single-incision anterior approach for total hip arthroplasty on an orthopaedic table. Clin Orthop Relat Res.

[6] Nakata K, Nishikawa M, Yamamoto K, Hirota S, Yoshikawa H (2009). A clinical comparative study of the direct anterior with mini-posterior approach: two consecutive series. J Arthroplasty.

[7] Judet J, Judet H (1985). Anterior approach in total hip arthroplasty. Presse Med.

[8] Laude F (2006). Total hip arthroplasty through an anterior Hueter minimally invasive approach. Interact Surg.

[9] Sariali E, Leonard P, Mamoudy P (2008). Dislocation after total hip arthroplasty using Hueter anterior approach. J Arthroplasty.

[10] Bhandari M, Matta JM, Dodgin D, Clark C, Kregor P, Bradley G, Little L, Anterior Total Hip Arthroplasty Collaborative Investigators (2009). Outcomes following the single-incision anterior approach to total hip arthroplasty: a multicenter observational study. Orthop Clin North Am.

[11] Hartford JM, Knowles SB (2016). Risk factors for perioperative femoral fractures: cementless femoral implants and the direct anterior approach using a fracture table. J Arthroplasty.

[12] Jewett BA, Collis DK (2011). High complication rate with anterior total hip arthroplasties on a fracture table. Clin Orthop Relat Res.

[13] Barrett WP, Turner SE, Leopold JP (2013). Prospective randomized study of direct anterior vs postero-lateral approach for total hip arthroplasty. J Arthroplasty.

[14] de Steiger RN, Lorimer M, Solomon M (2015). What is the learning curve for the anterior approach for total hip arthroplasty?. Clin Orthop Relat Res.

[15] Oinuma K, Eingartner C, Saito Y, Shiratsuchi H (2007). Total hip arthroplasty by a minimally invasive, direct anterior approach. Oper Orthop Traumatol.

[16] Restrepo C, Parvizi J, Pour AE, Hozack WJ (2010). Prospective randomized study of two surgical approaches for total hip arthroplasty. J Arthroplasty.

[17] Seng BE, Berend KR, Ajluni AF, Lombardi AV (2009). Anterior-supine minimally invasive total hip arthroplasty: defining the learning curve. Orthop Clin North Am.

[18] Homma Y, Baba T, Kobayashi H, Desroches A, Ozaki Y, Ochi H, Matsumoto M, Yuasa T, Kaneko K. Safety in early experience with a direct anterior approach using fluoroscopic guidance with manual leg control for primary total hip arthroplasty: a consecutive one hundred and twenty case series. Int Orthop. 2016 Mar;19 [Epub ahead of print]. 10.1007/s00264-016-3159-6 26993647

[19] ASA Physical Status Classification System. https://www.asahq.org/resources/clinical-information/asa-physical-status-classification-system. Accessed 29 Dec 2016.

[20] Gross JB (1983). Estimating allowable blood loss: corrected for dilution. Anesthesiology.

[21] Lewinnek GE, Lewis JL, Tarr R, Compere CL, Zimmerman JR (1978). Dislocations after total hip-replacement arthroplasties. J Bone Joint Surg Am.

[22] Kuribayashi M, Takahashi KA, Fujioka M, Ueshima K, Inoue S, Kubo T (2010). Reliability and validity of the Japanese Orthopaedic Association hip score. J Orthop Sci.

[23] Woolson ST, Pouliot MA, Huddleston JI (2009). Primary total hip arthroplasty using an anterior approach and a fracture table: short-term results from a community hospital. J Arthroplasty.

[24] Spaans AJ, van den Hout JA, Bolder SB (2012). High complication rate in the early experience of minimally invasive total hip arthroplasty by the direct anterior approach. Acta Orthop.

[25] Kobayashi H, Homma Y, Baba T, Ochi H, Matsumoto M, Yuasa T, Kaneko K (2016). Surgeons changing the approach for total hip arthroplasty from posterior to direct anterior with fluoroscopy should consider potential excessive cup anteversion and flexion implantation of the stem in their early experience. Int Orthop.

[26] Tamaki T, Nimura A, Oinuma K, Shiratsuchi H, Iida S, Akita K (2014). An anatomic study of the impressions on the greater trochanter: bony geometry indicates the alignment of the short external rotator muscles. J Arthroplasty.

[27] Ito Y, Matsushita I, Watanabe H, Kimura T (2012). Anatomic mapping of short external rotators shows the limit of their preservation during total hip arthroplasty. Clin Orthop Relat Res.

[28] Matsuura M, Ohashi H, Okamoto Y, Inori F, Okajima Y (2010). Elevation of the femur in THA through a direct anterior approach: cadaver and clinical studies. Clin Orthop Relat Res.

[29] Eto S, Hwang K, Huddleston JI, Amanatullah DF, Maloney WJ, Goodman SB. The Direct Anterior Approach is Associated With Early Revision Total Hip Arthroplasty. J Arthroplasty. 2016. doi: 10.1016/j.arth.2016.09.012. [Epub ahead of print] 10.1016/j.arth.2016.09.012 27843039

